# Respiratory Syncytial Virus Uses CX3CR1 as a Receptor on Primary Human Airway Epithelial Cultures

**DOI:** 10.1371/journal.ppat.1005318

**Published:** 2015-12-11

**Authors:** Sara M. Johnson, Beth A. McNally, Ioannis Ioannidis, Emilio Flano, Michael N. Teng, Antonius G. Oomens, Edward E. Walsh, Mark E. Peeples

**Affiliations:** 1 Center for Vaccines and Immunity, The Research Institute at Nationwide Children’s Hospital, Columbus, Ohio, United States of America; 2 Department of Pediatrics, The Ohio State University College of Medicine, Columbus, Ohio, United States of America; 3 Division of Allergy and Immunology, Department of Internal Medicine, Morsani College of Medicine, University of South Florida, Tampa, Florida, United States of America; 4 Department of Veterinary Pathobiology, Oklahoma State University, Stillwater, Oklahoma, United States of America; 5 School of Medicine and Dentistry, University of Rochester Medical Center, Rochester, New York, United States of America; University of North Carolina at Chapel Hill, UNITED STATES

## Abstract

Respiratory syncytial virus (RSV) is the most frequent cause of lower respiratory disease in infants, but no vaccine or effective therapy is available. The initiation of RSV infection of immortalized cells is largely dependent on cell surface heparan sulfate (HS), a receptor for the RSV attachment (G) glycoprotein in immortalized cells. However, RSV infects the ciliated cells in primary well differentiated human airway epithelial (HAE) cultures via the apical surface, but HS is not detectable on this surface. Here we show that soluble HS inhibits infection of immortalized cells, but not HAE cultures, confirming that HS is not the receptor on HAE cultures. Conversely, a “non-neutralizing” monoclonal antibody against the G protein that does not block RSV infection of immortalized cells, does inhibit infection of HAE cultures. This antibody was previously shown to block the interaction between the G protein and the chemokine receptor CX3CR1 and we have mapped the binding site for this antibody to the CX3C motif and its surrounding region in the G protein. We show that CX3CR1 is present on the apical surface of ciliated cells in HAE cultures and especially on the cilia. RSV infection of HAE cultures is reduced by an antibody against CX3CR1 and by mutations in the G protein CX3C motif. Additionally, mice lacking CX3CR1 are less susceptible to RSV infection. These findings demonstrate that RSV uses CX3CR1 as a cellular receptor on HAE cultures and highlight the importance of using a physiologically relevant model to study virus entry and antibody neutralization.

## Author Summary

Respiratory syncytial virus (RSV) is the second most common infectious cause of infant death worldwide. Despite this great clinical impact, no effective antivirals or vaccines against RSV are available. Here we find that the RSV attachment (G) glycoprotein uses CX3CR1 as a receptor on primary human airway epithelial (HAE) cultures, an excellent model of RSV infection of the human lung. The G protein contains a CX3C motif and we find that this region is critical for its role in infection of HAE cultures, but not of immortalized cells. Furthermore, we find that antibodies against the G protein neutralize RSV infection of HAE cultures differently from immortalized cells. These insights suggest that HAE cultures should be used to quantify neutralizing antibodies, including during vaccine development, that the CX3CR1 interaction with the RSV G protein could be a target for antiviral drug development, and that the G protein should be considered for inclusion in vaccines.

## Introduction

Respiratory syncytial virus (RSV) infects nearly every child by the age of 2 [1]. It causes severe lower respiratory disease in ~2% of these infants, making RSV infection the most frequent cause of hospitalization of infants and children in the developed world [2–4]. While supportive care successfully treats nearly all of these infants, in the developing world RSV infection causes the death of an estimated 66,000 to 199,000 children under five years of age annually [5,6]. The elderly are also susceptible to RSV disease and RSV is the second most frequent cause of ‘excess deaths’ during the winter months in this population, behind influenza virus [7,8]. Despite this great clinical impact, there are currently no approved vaccines or therapeutic antiviral drugs against RSV.

RSV infection has been studied mainly in immortalized cell lines, where the virion G glycoprotein uses cell-surface heparan sulfate as a receptor (HS) [9–11]. However, immortalized cell lines may not be the best model for the study of RSV entry as they differ in many aspects from the human airway epithelium *in vivo*. Primary, well differentiated human airway epithelial (HAE) cultures have been shown to accurately represent the human airway epithelium, both in appearance and function [12]. HAE cultures have served as a model for numerous respiratory viruses, including RSV, parainfluenza viruses, human/avian influenza viruses, and coronaviruses, and are considered to be an ideal *in vitro* model of viral interaction with the respiratory epithelium *in vivo* [13–18].

We previously found that RSV infects HAE cultures via the apical surface and nearly exclusively infects ciliated cells [19]. However, HAE cultures do not express detectable HS on their apical surface [13], leading us to hypothesize that a different viral receptor is responsible for RSV attachment to these cells and likely to human airways. CX3CR1, surfactant protein A, and annexin II have also been shown to bind the G protein and proposed to act as cellular receptors for RSV [20–23]. Recombinant RSV lacking its G gene is able to infect HAE cultures [24], albeit poorly, suggesting that the RSV F protein also has attachment activity. ICAM-1, TLR4, and nucleolin have been proposed to function as F protein receptors [25–27], but most of this work has been performed in immortalized cells and needs to be reexamined in primary cultures.

Here we compared the abilities of soluble HS and two anti-G monoclonal antibodies (mAbs) to inhibit RSV infection, finding that HS neutralized infection of HeLa cells but not HAE cultures and that the mAbs neutralized infection of HAE cultures much better than HeLa cells, indicating the use of different receptors on these different cells. One of the mAbs, 131-2g, previously characterized as “non-neutralizing” in immortalized cells, did neutralize RSV on HAE cells. This mAb had been shown to block G protein binding to CX3CR1 [23]. Here we find that CX3CR1 is detectable on ciliated cells in HAE cultures. Both a mAb against CX3CR1 and mutations in the G protein CX3C motif reduced RSV infection of these cells. These results suggest that RSV uses CX3CR1 as a cellular receptor on physiologically relevant HAE cultures and, likely, in the airways of the human lung.

## Materials and Methods

### Viruses and cells

Recombinant green fluorescence protein (GFP)-expressing RSV (rgRSV) (derived from D53, strain A2 with a variant F protein) [28] and a mutant of this virus lacking the genes encoding the G and SH proteins (rgRSV-F) which grows to lower titers than the parent virus [29], were previously described. The following reagent was obtained through BEI Resources, NIAID, NIH: Human Respiratory Syncytial Virus, A2001/2-20 (2–20), Purified from HEp-2 Cells, NR-43938. RSV strain B1 (subgroup B) was purchased from the American Type Culture Collection (Manassas, VA). MAb L9-resistant mutants RSV2 (F165L, F170L, I175T and C186R) and RSV6a (F168S, F170P, C186R and V225A) were selected as previously described from the WT (Long strain) of RSV [30]. Recombinant RSV D53 with a cysteine to serine mutation at amino acid 186 in the G protein (C186S RSV) was generated by reverse genetics [31], as was the RSV A2 CX3C-mutant strain with an alanine insertion at position 286 (CX4C RSV) [32]. All viruses were propagated and titered in HeLa cells (American Type Culture Collection). HeLa cells and 293A cells (American Type Culture Collection) were grown in DMEM containing 10% heat inactivated fetal bovine serum (FBS). Chinese hamster ovary (CHO) mutant A745 cells (gift from Dr. J.D. Esko, University of California, San Diego, San Diego, CA) which are severely deficient in the production of all glycosaminoglycans [33] were grown in RPMI 10% FBS.

Primary, well-differentiated human airway epithelial (HAE) cultures were grown on collagen coated Transwell inserts (Corning Incorporated, Corning, NY) as previously described [34]. Upon reaching confluency and forming tight junctions, the apical medium was removed and cultures were maintained at the air-liquid interface for 4 to 6 weeks to form well-differentiated, polarized cultures.

### Inoculation of HAE cultures

For neutralization experiments, rgRSV was incubated with increasing concentrations of heparan sulfate (Sigma-Aldrich, St. Louis, MO), protein A-purified mAb L9 [35] or mAb 131-2g [36] (gift from Dr. L.J. Anderson, Emory University School of Medicine, Atlanta, GA), compared to the appropriate mouse isotype controls, IgG_2a_ or IgG_1_ (R&D Systems, Minneapolis, MN). RSV isolate 2–20 or B1 were incubated with 5 μg/ml mAb 131-2g. All mAbs were diluted in DMEM 10% FBS and incubated with virus for 30 min at room temperature prior to inoculation of HeLa and HAE cultures. For anti-CX3CR1 blockade experiments, cells were incubated with 25 μg/ml CX3CR1 specific rat mAb (clone 2A9-1) (MBL International Corporation, Woburn, MA) or rat IgG_2b_ (Becton Dickinson, East Rutherford, NJ) for 30 min prior to inoculation. HeLa and HAE cultures were inoculated with ~200 PFU at 37°C. The inoculum was removed 2 hr later and cultures rinsed 3 times with PBS. At 24 hr post inoculation, cultures were fixed, permeabilized and incubated with an FITC-labeled anti-RSV polyclonal antibody (Virostat, Portland, ME). Infected (green from GFP or FITC) cells were detected and counted on an EVOS FL Cell Imaging System (Life Technologies, Carlsbad, CA).

### Construction and detection of G protein mutants

A codon-optimized version of the RSV A2 G protein gene (MP341) was modified to express the F120A mutant by inserting a synthetic gBlock Gene Fragment (Integrated DNA Technologies, Coralville, IA) into restriction digested parent plasmid. 293A cells were transfected with pcDNA3.1, MP341 or F170A using Lipofectamine 2000 (Life Technologies), or infected with rgRSV, WT (Long strain), RSV2, RSV6a or C186S viruses (MOI of 0.1). 24 hr post transfection/infection, cells were disrupted with Triton-X100, boiled, separated under reducing conditions by SDS-PAGE and transferred to nitrocellulose. The A2 strain RSV G protein was detected by immunoblot with 130-2g (gift from Dr. L.J. Anderson), L9, or 131-2g and anti-mouse antibody-horseradish peroxidase (HRP) (Kirkegaard & Perry Laboratories, Gaithersburg, MD). The RSV G protein from Long strain derived viruses was detected by immunoblot with rabbit antiserum against RSV (gift from Dr. P.L. Collins, NIAID, NIH, Bethesda, MD) and anti-rabbit antibody-HRP (Kirkegaard & Perry Laboratories) or L9 or 131-2g and anti-mouse antibody-HRP.

### Immunohistochemical analysis of HAE cultures

Cross-sections of formalin fixed and paraffin embedded HAE cultures were subjected to antigen retrieval (BioGenex, Fremont, CA) prior to incubation with polyclonal rabbit CX3CR1-specific IgG antibody ab8020 (Abcam, Cambridge, UK) or isotype control ab27478 (Abcam) followed by Texas Red labeled anti-rabbit IgG (Vector Laboratories, Burlingame, CA) and ProLong Gold anti-fade reagent with DAPI (Life Technologies, Carsbad, CA). Images were taken on an Olympus BX61 microscope (Olympus Corporation, Tokyo, Japan).

### Transfection and infection of CHO A745 cells

CHO A745 cells were transfected with a plasmid encoding human CX3CR1 (gift from Dr. P.M. Murphy, National Institute of Allergy and Infectious Disease) or pcDNA3.1 using Lipofectamine LTX (Life Technologies). 24 hr post transfection, cells were stained with a PE-labeled polyclonal anti-CX3CR1 antibody (BioLegend, San Diego, CA) and imaged on an EVOS FL Cell Imaging System (Life Technologies) to determine cell surface expression. At 24 hr post transfection, parallel wells of cells were inoculated as above with rgRSV at an MOI of 0.1. 48 hr post inoculation, infected (green fluorescent) cells were visualized and counted.

### *In vivo* assays

C57BL/6 CX3CR1GFP mice, which lack functional CX3CR1 through replacement of the murine CX3CR1 gene with that for enhanced green fluorescent protein [37], and C57BL/6 mice (gift from Dr. S. Partida-Sanchez, Nationwide Children’s Hospital, Columbus, OH) were maintained in biosafety level 2 (BL2) containment under pathogen-free conditions.

Male mice (age, 6 to 8 wk) were lightly anesthetized and inoculated intranasally (i.n.) with 10^6^ PFU of rgRSV or 10^5^ PFU of rgRSV-F in a 30 μl volume. No animals were excluded from analyses. No blinding was done. At 5 days post inoculation, 3 mice per group were examined. Lungs were harvested and homogenized and viral titers were assayed on HeLa cells.

### Ethics statement

All animal experiments were carried out in strict accordance with the accredited conditions in the Guide for the Care and Use of Laboratory Animals of the National Institutes of Health. The protocol was approved by the Institutional Animal Care and Use Committee (Welfare Assurance Number A3544-01) at The Research Institute at Nationwide Children's Hospital, AR09-00012.

All experimental procedures were performed under isoflurane anesthesia, and all efforts were made to minimize suffering.

### Statistical analyses

All data presented for *in vitro* experiments are a single experiment representative of three. Data from three replicates of each experimental condition are expressed as mean ± standard deviation. Data for *in vivo* experiments with rgRSV are expressed as the mean ± standard deviation of three combined experiments (each with three animals per group). All experiments were repeated ≥ 3 times. A 2-tailed student’s t-test was employed to determine the significance of the differences between experimental conditions. A P-value of <0.05 was considered to be statistically significant.

## Results

### Soluble HS efficiently blocks RSV infection of HeLa, but not HAE cells

**Fig 1:**
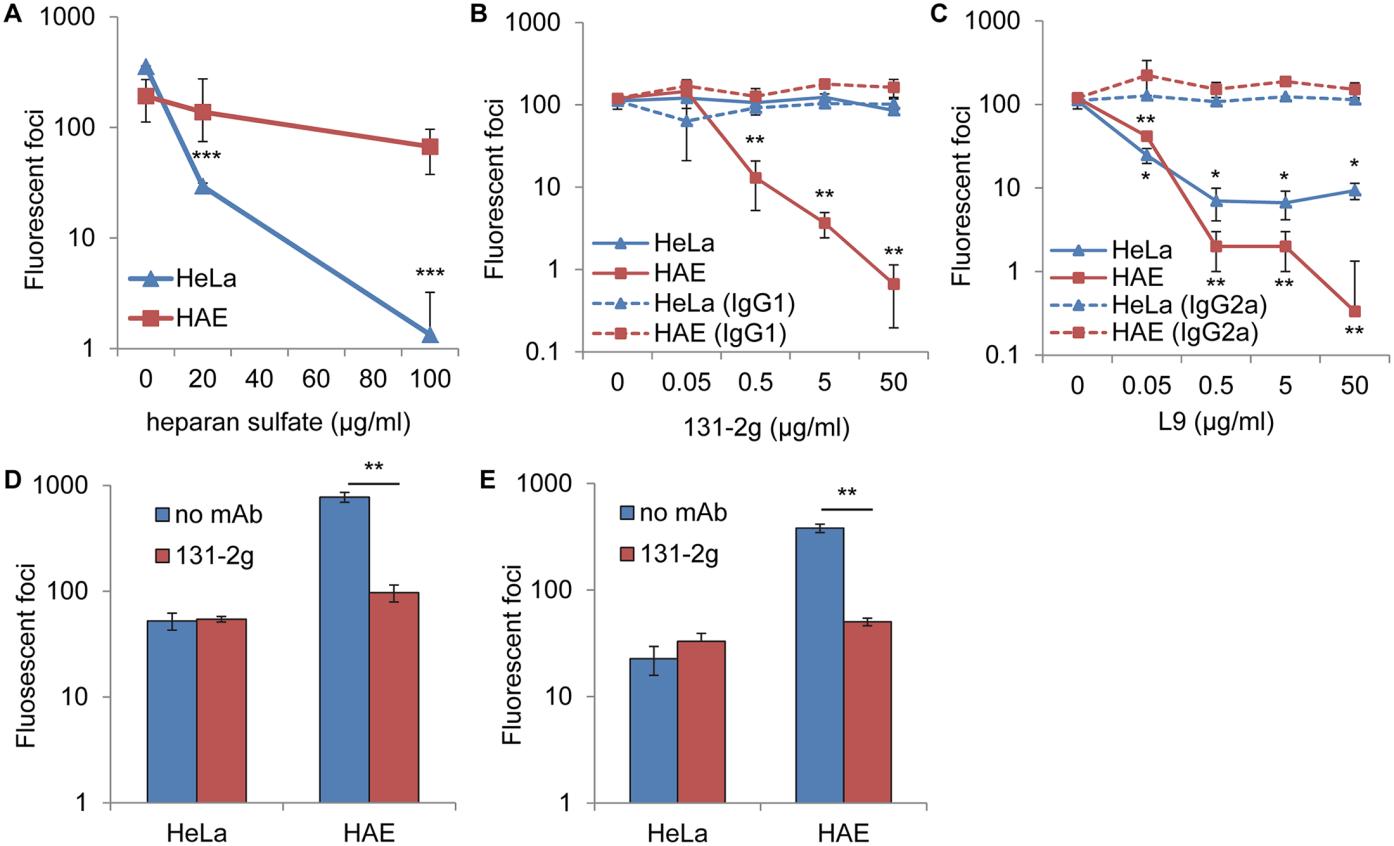
Differential neutralization of RSV infection of HeLa and HAE cultures. rgRSV was incubated with (A) soluble HS, (B) anti-G protein mAb 131-2g or IgG1 (isotype control), or (C) anti-G protein mAb L9 or IgG2a for 30 min prior to inoculation of HeLa and HAE cultures. (D) 2–20 or (E) B1 was incubated with 5 μg/ml mAb 131-2g for 30 min prior to inoculation of HeLa and HAE cultures. Infected cells (fluorescent foci) were counted at 24 hr post inoculation. All data are of one experiment representative of three. Data are expressed as the mean ± s.d. of 3 wells. Two-sided student's *t* test: *P < 0.05, **P < 0.01, ***P < 0.001.

HS is not detectable on the apical surface of well-differentiated HAE cultures [13], suggesting that RSV uses a receptor other than HS to attach to these physiologically relevant cells. If HS is not the virus receptor on HAE cells, soluble HS should not reduce RSV infection of these cells. To test this possibility, recombinant green fluorescent protein expressing RSV (rgRSV) was incubated with soluble HS prior to inoculation of HeLa and HAE cultures. Soluble HS (20 μg/ml) neutralized infection of HeLa cells without reducing infection of HAE cultures (Fig 1A). This result is consistent with HS acting as a virus receptor on HeLa cells, but not on HAE cultures. At a 5-fold higher concentration of soluble HS (100 μg/ml) RSV infection of HeLa cultures was reduced by over 100-fold, but infection of HAE cultures was reduced by only 2-fold. The partial sensitivity of RSV to this higher concentration may be the result of HS binding to lower affinity sites on the virions.

### Two mAbs against the G protein neutralize RSV infection of HAE cultures much more effectively than in HeLa cells

If RSV uses a receptor other than HS to attach to HAE cultures, monoclonal antibodies (mAbs) against the G protein may differ in their ability to block virus attachment and entry into immortalized versus HAE cultures depending on where on the G protein they bind. MAb 131-2g binds the G protein and has been characterized as “non-neutralizing” because it does not reduce RSV infection of HEp-2 cells [36] and we confirmed this result for HeLa cells (Fig 1B). However, mAb 131-2g did neutralize rgRSV infection of HAE cultures. The ability of mAb 131-2g to reduce infection of HAE, but not HeLa cultures suggests that mAb 131-2g binds near and interferes with the function of a domain in the G protein that is important for attachment to HAE cultures but not for attachment to HeLa cells. This result demonstrates that these two domains on the G protein are distinct and confirms our hypothesis that RSV uses a different receptor to infect HAE cells compared to HeLa cells. Furthermore, these results indicate the importance of using physiologically relevant HAE cultures for determining the neutralizing abilities of antibodies against the G protein.

We also tested G protein mAb L9 [35] for its ability to neutralize rgRSV. MAb L9 was previously shown to neutralize RSV infection of immortalized cells [35,38]. However, we found that mAb L9 also inhibited rgRSV infection of HAE cells, but the inhibition of HAE infection was approximately 10-fold greater than the inhibition of HeLa infection (Fig 1C). The ability of mAb L9 to neutralize rgRSV on HeLa and HAE cultures may be because the mAb blocks G protein binding to both HS and the unidentified HAE receptor, respectively. We have previously found that recombinant RSV lacking the G gene is much less infectious for HAE cultures than for HeLa cells [24], indicating that the G protein is more important for infection of HAE cultures. The greater neutralizing ability of mAb L9 on HAE cultures is likely due to the fact that the G protein is more critical for efficient infection of these cells. Therefore, the ability of mAb L9 to neutralize rgRSV infection of both HeLa and HAE cells suggests that the attachment sites on the G protein for each cell type are close to each other.

For other paramyxoviruses, receptor binding differs between laboratory adapted strains and clinical isolates [39,40]. To confirm that mAb 131-2g neutralization of rgRSV (subgroup A) in HAE cultures is not an artifact of using a laboratory adapted virus, we examined the neutralizing ability of this mAb for a low-passage subgroup A clinical isolate, RSV A2001/2-20 (2–20) [41]. MAb 131-2g efficiently neutralized 2–20 infection of HAE cultures, but not of HeLa cells (Fig 1D), similar to rgRSV. We also examined the neutralizing ability of mAb 131-2g for B1, an member of the RSV subgroup B. B1 infection of HAE cultures was also reduced, while infection of HeLa cells was not, similar to rgRSV (Fig 1E). These results demonstrate that regardless of the source or subgroup of RSV, mAb 131-2g neutralizes infection of HAE cultures, but not HeLa cells.

### The G protein epitope recognized by mAb 131-2g includes C186, part of the CX3C motif

**Fig 2:**
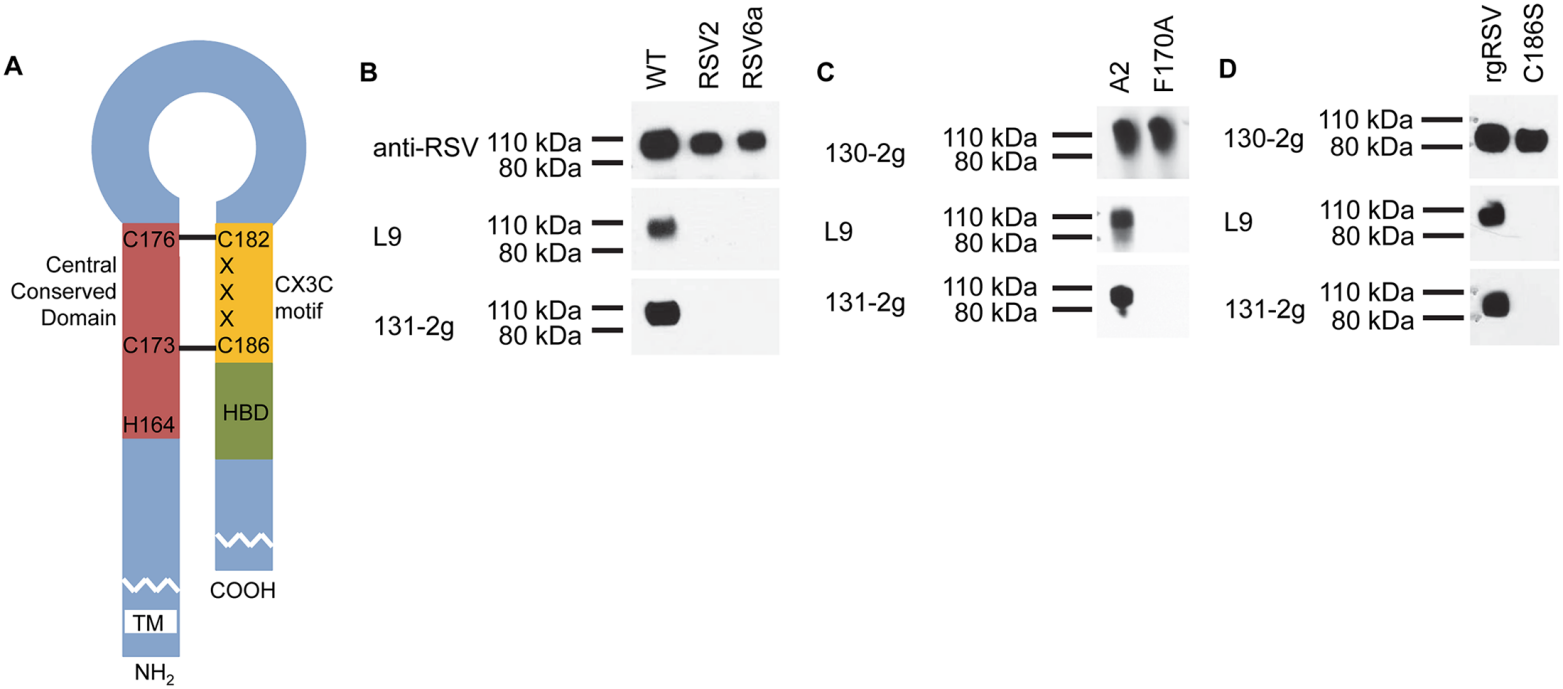
MAb binding to the RSV G protein. (A) The G protein, an N-terminally anchored [37] type II glycoprotein, has a central conserved region that overlaps with the cysteine noose held together by two disulfide bonds. The third and fourth cysteines are also part of the CX3C motif. C terminal to the CX3C motif is a highly basic heparin binding domain (HBD). RSV G protein (90 kDa) from (B) parental WT (Long strain) RSV and L9 neutralization escape mutants RSV2 (F165L, F170L, I175T and C186R) and RSV6a (F168S, F170P, C186R and V225A), (C) parental strain A2 RSV and recombinant RSV with a F170A mutation, or (D) parental strain A2 RSV and recombinant RSV with a C186S mutation were detected by immunoblot with the indicated antibodies.

The RSV G protein is an N-terminally anchored type II transmembrane protein [42] (Fig 2A). It has a highly conserved central domain that is flanked by two highly O-glycosylated, mucin-like regions that also contain several N-linked glycans [42,43]. Partially overlapping this central conserved region are 4 conserved cysteines linked by two disulfide bonds to form the neck of a ‘noose’ [44]. The last 2 cysteines are part of a CX3C motif [23]. Immediately C-terminal to this motif is the highly basic heparin-binding domain that binds to HS [45].

MAb L9 has been used to select neutralization-resistant viruses in HEp-2 cells. These resistant mutants each have multiple amino acid substitutions clustered in and around the central conserved domain of the G protein indicating that L9 binds to this region [30]. MAb 131-2g binds a fragment of the G protein that includes amino acids 1 to 173 and reacts with both RSV subtypes, A and B [46]. The amino acid sequence of this fragment is less than 44% conserved between the strains with the exception of the completely conserved 164–176 region. These observations suggest that mAb 131-2g, like mAb L9, binds within the central conserved domain of the G protein. Because mAb 131-2g is non-neutralizing in immortalized cells, no neutralization resistant virus variants have been isolated.

To determine if mAb 131-2g binds at or near the same region as mAb L9, we used immunoblotting of the G protein in parental Long strain RSV (WT) and two L9-selected RSV mutants derived from it, RSV2 and RSV6a. Partially purified virions from these mutants contain the G protein, detectable with rabbit anti-RSV serum, as does WT (Fig 2B). As expected, mAb L9 detected the WT RSV G protein, but not the G protein from the two L9 neutralization-resistant RSV mutants. MAb 131-2g was also able to detect WT RSV, but not the two L9 neutralization-resistant RSV mutants, suggesting that one or more of the mutated amino acids in RSV2 and RSV6a are also important for mAb 131-2g binding.

Both mAb L9-resistant RSV mutants contain multiple mutations in their G proteins: F165L, F170L, I175T and C186R in RSV2; and F168S, F170P, C186R and V225A in RSV6a [30]. Both have mutations in F170 and C186. To determine if F170 is required for mAb L9 and 131-2g binding, we generated a plasmid encoding a G protein with an F170A mutation and tested the reactivity of each mAb to this mutant G protein by immunoblot. Neither mAb was able to detect the F170A mutant G protein (Fig 2C). Mutant G protein was present in the virions as demonstrated by another mAb, 130-2g, which binds within the C-terminal region of the G protein.

To determine if C186 is required for mAb L9 and 131-2g binding we generated a recombinant RSV whose only mutation is C186S in its G protein and tested the ability of these mAbs to bind. Virions from the C186S mutant did contain G protein as detected by mAb 130-2g, but neither mAb L9 nor 131-2g were able to detect the mutant G protein (Fig 2D). These results indicate that both F170 and C186 are critical for both mAb L9 and 131-2g binding to the G protein and, therefore, that the epitopes of these mAbs overlap. However, as shown above, mAb L9 neutralizes RSV infection of HAE cultures more efficiently than mAb 131-2g and neutralizes RSV in HeLa cells, unlike 131-2g (Fig 1B and 1C), indicating that while the epitopes of these mAbs overlap, they are not identical.

### CX3CR1 is detectable on the apical surface of ciliated HAE cells

**Fig 3:**
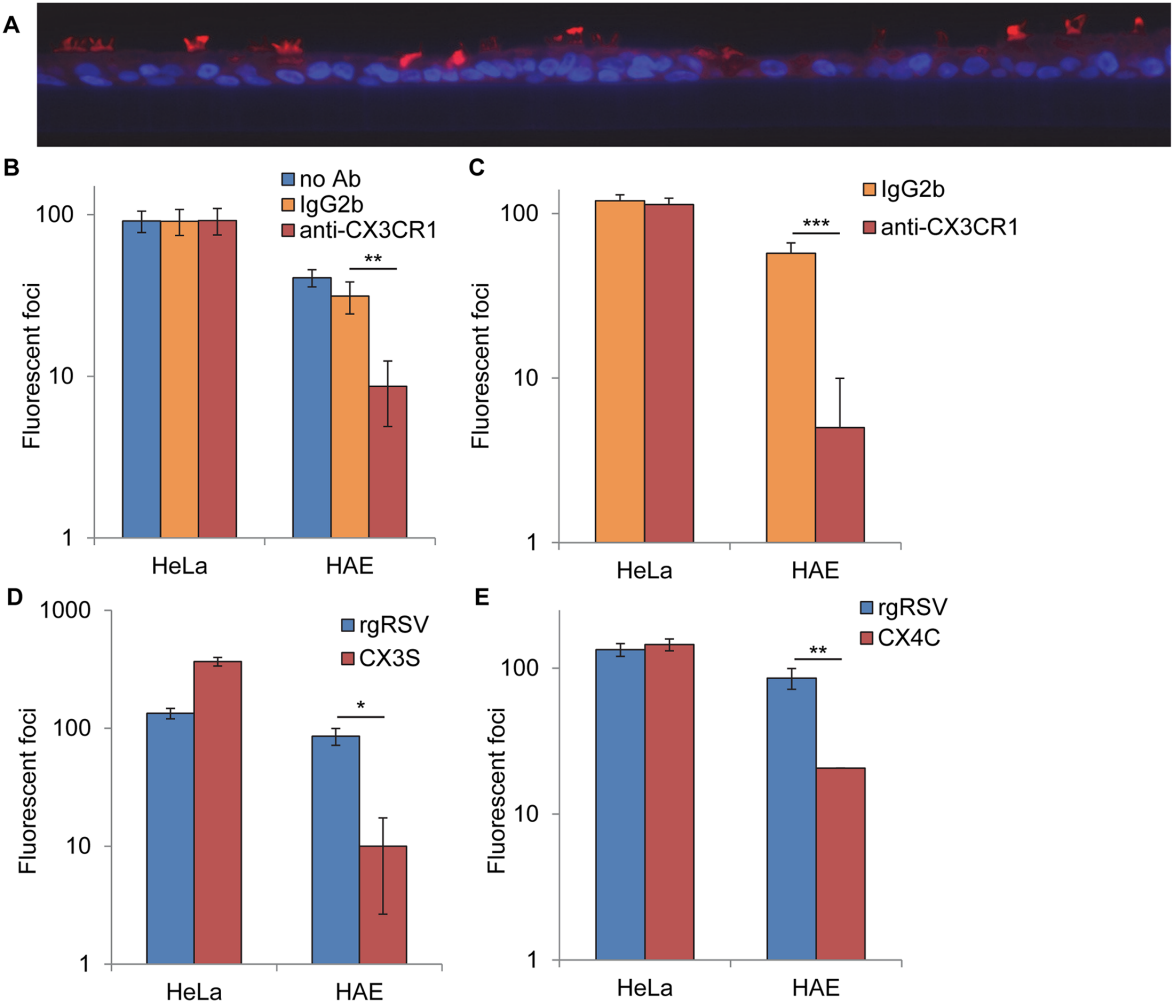
Detection of CX3CR1 on HAE cultures and importance of the RSV G protein interaction with CX3CR1 for infection. (A) Cross-section of a paraffin embedded HAE cultures stained with polyclonal antibodies to human CX3CR1 (red) and counterstained with DAPI to identify nuclei (blue). Images were taken with a 20X objective. (B) HeLa and HAE cultures were incubated with DMEM, 25 μg/ml isotype control, or 25 μg/ml anti-CX3CR1 for 30 min prior to inoculation with rgRSV. (C) Cells were incubated with 25 μg/ml anti-CX3CR1 or isotype control before, during and after apical inoculation with rgRSV. (D) HeLa and HAE cultures were inoculated with rgRSV or C186S RSV or (E) with rgRSV or CX4C RSV. Infected cells (fluorescent foci) were counted at 24 hr post inoculation. All data are of one experiment representative of three. Data from b-d are expressed as the mean ± s.d. of 3 wells. Two-sided student's *t* test: *P < 0.05, **P < 0.01, ***P < 0.005.

C186 is the second cysteine of the CX3C motif in the G protein. The cysteine spacing of this motif is similar to the only CX3C chemokine, fractalkine [23]. The G protein has been shown to bind to the fractalkine receptor, CX3CR1, and can use it to initiate infection when it is transiently expressed in immortalized cells [23]. MAb 131-2g blocks G protein binding to CX3CR1 [23]. Here we found that mAb 131-2g efficiently reduces RSV infection of HAE cultures (Fig 1b–1e), leading us to hypothesize that RSV uses CX3CR1 as a receptor on these cells. To determine if CX3CR1 is expressed on HAE cells in general and on ciliated cells, the target cells for RSV infection, we stained fixed cross-sections of HAE cultures with antibodies against CX3CR1. These antibodies stained the apical surface of HAE cultures, primarily and robustly staining the cilia on ciliated cells (Fig 3A). This finding indicates that CX3CR1 is located on the correct cell type to be an RSV receptor on HAE cultures. Its expression in this location could be responsible for the ciliated cell tropism of RSV.

### The G protein CX3C motif is important for infection of HAE cultures

To validate the functional role of CX3CR1 on HAE cultures, we incubated HAE and HeLa cultures with CX3CR1-specific mAb or isotype control prior to inoculation with rgRSV. Anti-CX3CR1 reduced RSV infection of HAE cultures significantly compared to isotype control, while neither antibody reduced RSV infection of HeLa cells (Fig 3B). Incubation of HAE cultures with anti-CX3CR1 before, during and after inoculation inhibited RSV infection to a greater extent (Fig 3C), but again did not reduced infection of HeLa cultures. These results indicate that RSV interaction with CX3CR1 is important for efficient infection of HAE cultures, but not HeLa cells.

If CX3CR1 is a receptor for the RSV G protein on HAE cultures, the G protein CX3C motif would likely be important for infection of these cells. To test this possibility, we inoculated HeLa and HAE cultures with rgRSV or the RSV mutant described above with a mutation in the last cysteine of the CX3C motif (C186S RSV). rgRSV and C186S RSV were comparably infectious for HeLa cells (Fig 3D). In contrast, while rgRSV readily infected HAE cultures, C186S RSV was poorly infectious for these cells, similar to RSV completely lacking the G protein [24]. This finding indicates that C186 in the G protein is critical for RSV infection of HAE cultures and may be involved in RSV attachment to these cells.

Next, we determined the infectivity of recombinant RSV with an additional amino acid (alanine) inserted between the two cysteines in the CX3C motif, CX4C RSV. rgRSV with an intact CX3C motif was comparably infectious for HeLa and HAE cultures (Fig 3E). However, CX4C RSV was poorly infectious for HAE cultures. These results with both of these mutant viruses indicate that the CX3C motif is important for efficient infection of HAE cultures, supporting the hypothesis that CX3CR1 is a receptor on these cells.

### CX3CR1 is sufficient to act as a receptor in the absence of HS

The CHO A745 cell line is defective in xylosyl transferase, the enzyme that initiates glycosaminoglycan synthesis on a protein by linking xylose to a serine or threonine in the proper context [33]. The result of this defect is a severe deficiency in total glycosaminoglycan expression, including expression of HS. RSV is poorly infectious for CHO A745 cells, infecting them 17-fold less efficiently than CHO K1 cells [24].

**Fig 4:**
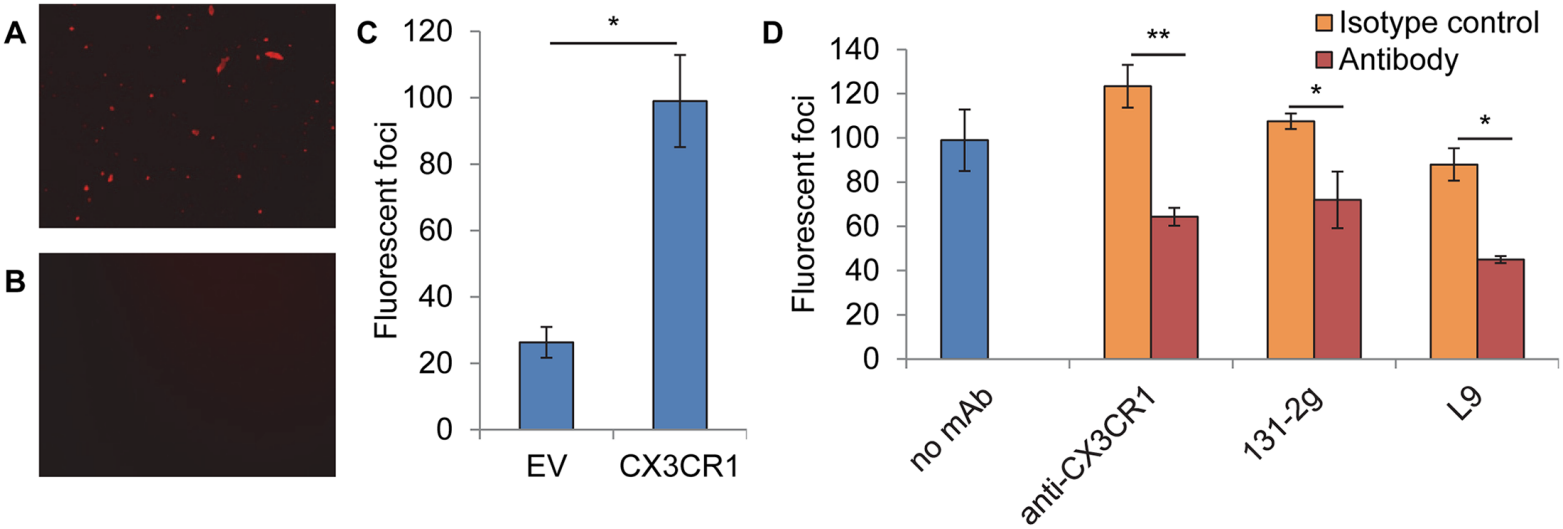
RSV infection of CHO A745 cells expressing CX3CR1. CHO A745 cells were transfected with a plasmid encoding (A) CX3CR1 or (B) empty vector and stained 24 hr later with anti-CX3CR1. Image was taken with a 20X objective. 24 hr post-transfection cells were inoculated with rgRSV in the (C) absence or (D) presence of 25 μg/ml anti-CX3CR1 or 5 μg/ml L9, 131-2g, or their isotype controls. Infected cells (fluorescent foci) were counted at 24 hr post inoculation. All data are of one experiment representative of three. Data in C and D are expressed as the mean ± s.d. of 3 wells. Two-sided student's *t* test: *P < 0.05, **P < 0.01.

To demonstrate that CX3CR1 is capable of acting as a receptor in the absence of HS, we transiently expressed CX3CR1 in CHO A745 cells, a cell line deficient in HS expression, and determined RSV infectivity for these cells. At the time of inoculation roughly 10% of cells expressed cell surface CX3CR1 (Fig 4A), while control cells did not (Fig 4B). Expression of CX3CR1 resulted in a 4-fold increase in infection as compared to the same cells transfected with empty vector (Fig 4C). A mAb against CX3CR1 significantly reduced RSV infection compared to an isotype control (Fig 4D), indicating that the increase in infection was due to RSV interaction with the receptor. Further, mAbs 131-2g and L9 significantly reduced RSV infection of CX3CR1 expressing cells, supporting the hypothesis that these mAbs neutralize RSV infection by blocking the interaction of the G protein with CX3CR1. Together, these data suggest that CX3CR1 is a receptor on HAE cultures and that the neutralizing ability of antibodies against the G protein can differ when assayed in cells expressing CX3CR1 rather than HS.

### CX3CR1 knockout mice are less susceptible to RSV infection than WT mice

**Fig 5:**
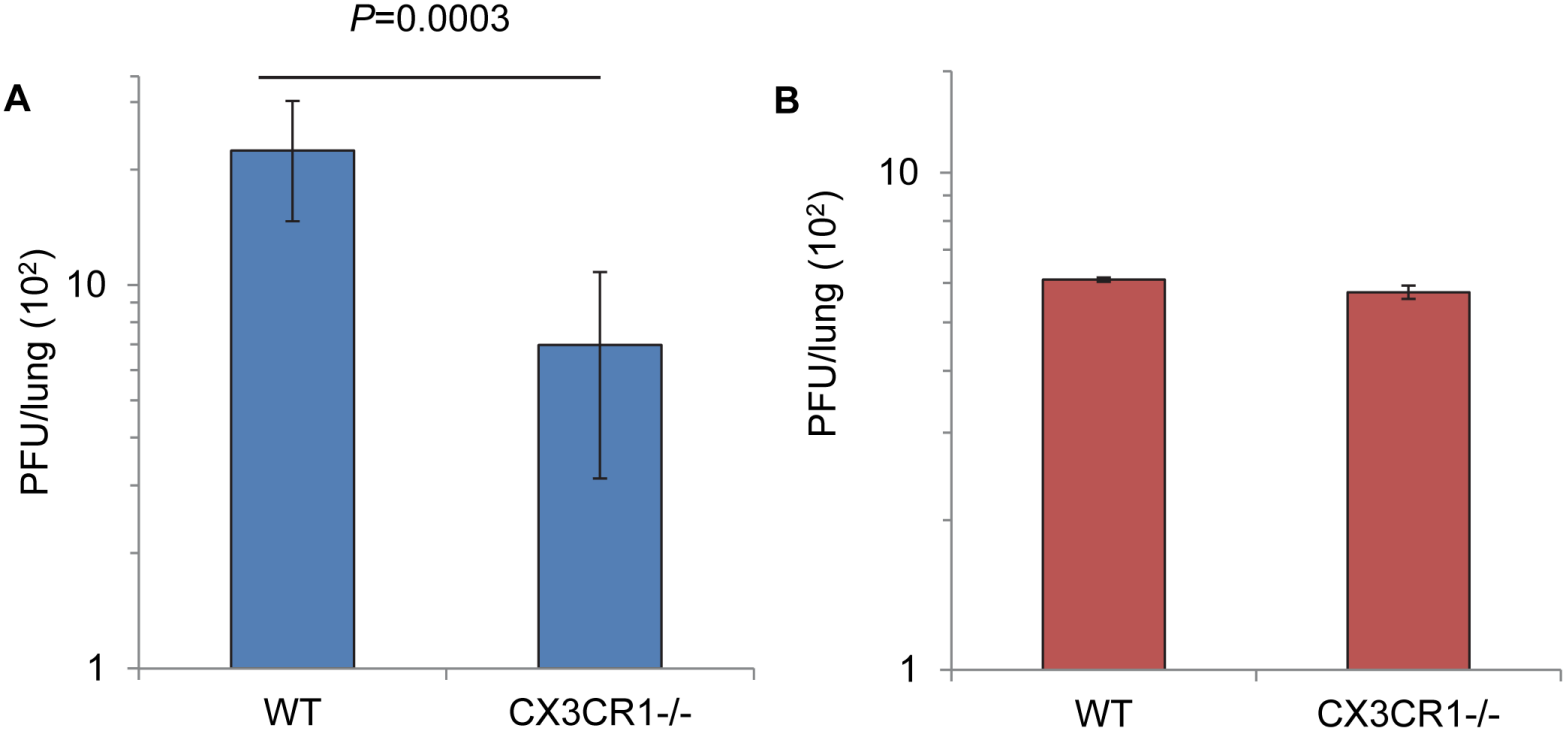
RSV infection of WT and CX3CR1-/- mice. For each experiment, WT and CX3CR1-/- mice (3 mice per group) were intranasally inoculated with (A) 10^6^ PFU rgRSV or (B) 10^5^ PFU rgRSV-F, which lacks the G and SH genes and grows to lower titers than rgRSV. Lungs were harvested at 5 dpi and PFU per lung was determined. Data for three rgRSV experiments were combined and expressed as the mean ± s.d. of 9 mice. The two-sided student's *t* test was used to determine significance.

To examine the importance of CX3CR1 as a receptor *in vivo*, we intranasally inoculated WT and CX3CR1-/- mice with rgRSV. Five days post inoculation, at the peak of RSV replication in the mouse lung, we found that CX3CR1-/- mice had significantly lower titers of RSV (Fig 5A) relative to WT mice. This result supports the hypothesis that murine CX3CR1 acts as an RSV receptor in the mouse lung.

In this experiment, rgRSV retained some of its infectivity for CX3CR1-/- mice, suggesting that RSV also uses an alternate, less efficient mechanism for initiating infection in the absence of the G protein-CX3CR1 interaction. To examine this possibility, we inoculated mice with rgRSV lacking the G and SH genes, such that the F protein is the only glycoprotein expressed, and found that this virus infected both WT and CX3CR1-/- mice comparably, albeit poorly (Fig 5B). This finding suggests that G protein interaction with CX3CR1 is important for efficient infection of mice, but that the F protein may also have some attachment activity.

## Discussion

RSV efficiently infects many immortalized cell lines, but it is not clear how closely this infectious process represents RSV infection of the human airway. The results in this report indicate that the initial, receptor-mediated step of infection in a primary cell culture model for the human airway epithelium, well-differentiated HAE cultures, differs from the initial step of infection in HeLa cells. The cellular receptor in immortalized cells is HS [9,10]. However, HS is not detectable on the apical surface of HAE cultures [13] and, as shown here, soluble HS does not inhibit infection of HAE cultures, indicating that a different receptor is used in these cultures and, therefore, *in vivo*. CX3CR1, surfactant protein A, and annexin II have been shown to bind the G protein and proposed to act as cellular receptors for RSV [20–23], but the functional *in vivo* receptor(s) for RSV had not been identified. Here we present evidence for CX3CR1 as a cellular receptor on physiologically relevant HAE cultures and, therefore, in the human lung.

Tripp et al. found that the RSV G protein mimics the chemokine CX3CL1 (fractalkine) in its ability to bind to its receptor, CX3CR1 [23]. CX3CR1 is expressed in epithelial cells, smooth muscle cells, microglia, neurons, T cells, monocytes, dendritic cells, and NK cells [47–53]. RSV is able to infect nearly all immortalized cell lines and infects primary epithelial cells, smooth muscle cells, neuronal cells, eosinophils, and dendritic cells [19,40,54,55]. Here we found that CX3CR1 is detectable on the cilia of ciliated cells in HAE cultures, the cell targeted by RSV [19]. In two publications that appeared while the present report was under review, Jeong et al. confirmed this location [56] and Chirkova et al. found CX3CR1 on the majority of RSV infected cells in primary human bronchial epithelial cultures [57].

MAb 131-2g has been shown to block G protein binding to CX3CR1 [23]. MAb 131-2g also reduces leukocyte migration *in vitro* [23] and inflammation in the lungs of RSV infected mice [58], suggesting that the G protein modulates the host inflammatory response via its interaction with CX3CR1. In RSV infected mice, treatment with mAb 131-2g mediates viral clearance and reduces RSV pathogenesis [59]. Because mAb 131-2g is non-neutralizing in immortalized cells, it was thought that this reduction in virus load could not be due to virus neutralization, but might instead be due to antibody dependent cell mediated cytotoxicity. Furthermore, it was suggested that the reduction in pathogenesis was due to prevention of G protein-mediated leukocyte chemotaxis. Here we demonstrate that mAb 131-2g is strongly neutralizing on HAE cultures and that CX3CR1 is likely an important receptor on these cells. As our CX3CR1-/- mouse infection data suggest that RSV uses CX3CR1 as a receptor on the murine airway epithelium, RSV neutralization by mAb 131-2g could have directly caused the decrease in viral load, resulting in decreased pathogenesis. A previous report did not find a difference in RSV yield from CX3CR1-/- and WT mice [60]. However, infectious virus in the lung was not quantified in that study which relied instead on real-time PCR. Indeed, several studies have utilized the mouse model and mAb 131-2g to examine the effects of the RSV G protein-CX3CR1 interaction on the immune response to RSV, but its use of CX3CR1 as a receptor on epithelial cells in the murine lung has not yet been directly examined.

Differences in measles virus (MV) receptor usage between clinical isolates and laboratory strains have been well documented to be due to selection during growth in immortalized cells. SLAM (CD150) is used as a receptor by clinical isolates [40], but not by the laboratory adapted Edmonston strain of MV which uses CD46 [56]. Because mAb 131-2g blocks G protein binding to CX3CR1, our finding that mAb 131-2g neutralizes infection of clinical isolates for HAE cultures (Fig 1D) indicates that the use of CX3CR1 is not an artifact of using a laboratory strain of RSV.

RSV infection of cells lacking CX3CR1 suggests that RSV interacts with an additional receptor on these cells. Recombinant RSV lacking the G gene is able to infect HAE cultures [24], albeit poorly, suggesting that there may also be an F protein receptor on these cultures. It is possible that more than one receptor may be involved in RSV infection of airway cells. Others have presented evidence that ICAM-1, TLR4, and nucleolin can function as F protein receptors [25–27]. These proposed RSV receptors have been studied mainly in immortalized cells and further investigation is needed to determine their role, if any, in RSV infection of HAE cultures.

Here we have localized a critical element of the binding site of 131-2g, a mAb that neutralizes RSV infection in HAE cultures, but not in HeLa cultures. We found that both F170 and C186 of the G protein are required for binding by mAbs L9 and 131-2g on an immunoblot, indicating that the epitopes of these mAbs overlap. Furthermore, since both mAbs bound the G protein that had been treated with a reducing agent, mAb binding was not dependent on the structure provided by the disulfide bonds, but rather on the primary sequence. Sullender previously found that mAb 131-2g does not bind to cells expressing a fragment of the G protein that includes amino acids 1 to 173 in an immunoblot [46], but does bind this fragment in a cell immunofluorescence assay. It is possible that the site on the G protein that is recognized by mAb 131-2g has two components, one that includes amino acids F170 and C186 and another that is conformational. Further characterization is required and may provide further insights into this HAE-only neutralizing epitope on the G protein.

Titers of serum neutralizing antibodies correlate with protection from RSV [61,62]. The findings presented here demonstrate that some antibodies to the G protein, one of only two neutralizing antigens in RSV, can neutralize RSV infection only when assessed on physiologically relevant HAE cultures. For this reason, some of the *in vivo* neutralizing antibodies to the G protein may be missed when assessing neutralizing activity on immortalized cells. Conversely, it is possible that other antibodies that neutralize RSV on immortalized cells may not have neutralizing activity *in vivo*. Moreover, it is possible that the use of HAE cultures may be important for more accurately quantifying neutralizing antibodies against other respiratory viruses, particularly those that use HS as a surrogate receptor for infection in immortalized cells.

The importance of using a physiologically relevant model to study paramyxovirus entry has been previously demonstrated. Palmer et al. found that fusion inhibitors against human parainfluenza virus (HPIV) display similar inhibition *in vivo* and in HAE cultures, but not in immortalized cell culture [39]. These findings demonstrate, and ours confirm, the importance of evaluating antivirals in natural host tissue.

Efficient neutralization of RSV infection of HAE cultures by mAbs against the G protein bolsters the suggestion that the G protein should be considered for inclusion in vaccine candidates. Indeed, vaccination of mice or cotton rats with a G protein fragment (amino acids 131–230) induces neutralizing antibodies and protects against RSV challenge [63,64]. Also, mice immunized with a shorter peptide (amino acids 148 to 198) generate antibodies that neutralize both A and B strains of RSV [65]. However, the neutralizing activity of these antibodies was determined using immortalized cells. Neutralizing activity determined on HAE cultures may well be greater and should more accurately reflect neutralizing activity *in vivo*.
